# Image identification from brain activity using the population receptive field model

**DOI:** 10.1371/journal.pone.0183295

**Published:** 2017-09-18

**Authors:** Wietske Zuiderbaan, Ben M. Harvey, Serge O. Dumoulin

**Affiliations:** 1 Experimental Psychology, Helmholtz Institute, Utrecht University, Utrecht, The Netherlands; 2 Spinoza Centre for Neuroimaging, Amsterdam, The Netherlands; University College London, UNITED KINGDOM

## Abstract

A goal of computational models is not only to explain experimental data but also to make new predictions. A current focus of computational neuroimaging is to predict features of the presented stimulus from measured brain signals. These computational neuroimaging approaches may be agnostic about the underlying neural processes or may be biologically inspired. Here, we use the biologically inspired population receptive field (pRF) approach to identify presented images from fMRI recordings of the visual cortex, using an explicit model of the underlying neural response selectivity. The advantage of the pRF-model is its simplicity: it is defined by a handful of parameters, which can be estimated from fMRI data that was collected within half an hour. Using 7T MRI, we measured responses elicited by different visual stimuli: (i) conventional pRF mapping stimuli, (ii) semi-random synthetic images and (iii) natural images. The pRF mapping stimuli were used to estimate the pRF-properties of each cortical location in early visual cortex. Next, we used these pRFs to identify which synthetic or natural images was presented to the subject from the fMRI responses. We show that image identification using V1 responses is far above chance, both for the synthetic and natural images. Thus, we can identify visual images, including natural images, using the most fundamental low-parameter pRF model estimated from conventional pRF mapping stimuli. This allows broader application of image identification.

## Introduction

Determining a human’s mental state from their measured brain activity is a great challenge of neuroscience. Different decoding techniques have been used to determine what the subject was seeing, hearing, remembering or dreaming by analyzing fMRI activation patterns (for review see: [1]. Most of these studies use machine-learning techniques, where a classifier is trained on a set of activation patterns elicited by known stimuli. The classifier then compares a new activation pattern to this trained set of activation patterns to predict the state of an unknown stimulus. For example, it is possible to train a classifier using brain activation patterns elicited by sets of house and face images [2]. That classifier can then distinguish whether a new activation pattern was elicited by a house or a face image. However, this approach can only make predictions about a state that the classifier was explicitly trained on: a classifier trained to distinguish mental states elicited by house and face stimuli cannot distinguish between those elicited by, for example, animal and tree stimuli.

A different approach to make predictions about visually presented stimuli uses biologically inspired encoding models. These models are based on the brain’s representation of stimuli [3–8], and reveal not only what stimulus state is represented in the brain by also how the stimulus is represented. Kay and colleagues (2008) use a Gabor Wavelet Pyramid (GWP) as a biologically inspired encoding model for the early visual system. The model uses a training set of known natural images to determine the properties on the GWP. They identify presented images outside this training set using brain activation patterns measured with fMRI, and the properties of the GWP. The model predicts brain activity based on the inherent features of the stimulus. Since the model simulates the computations of the brain, it can make predictions for new stimuli. It can therefore identify new images that the model was not explicitly trained on. The downside of the GWP is that it uses many free parameters to capture the neural responses (2370), and requires long fMRI scan times (five hours per subject) for training.

Here we ask whether image identification is also possible using a basic biologically inspired encoding model that is already used in many conventional vision studies, i.e. the pRF model [3]. The rationale of this study is two-fold. First, the study is a validation of the pRF-model on natural images. Using the pRF-model we can make a prediction of the measured brain activity to any image based on the contrast information of that image. Here we demonstrate that the representation of the image by the pRF-model corresponds to the actual representation based upon the measured brain activity in response to natural images. Second, this study allows a deeper insight into visual processing mechanisms operating on natural images. For example, we can evaluate if there are image features beyond contrast that are not included in the pRF-model but affect responses of visual cortex. So, where on the one hand this study shows us how contrast information is represented in V1, it also provides us information about the deviation of the representation in V1 from this contrast information.

The model was trained on responses to standard visual field mapping stimuli, which can be measured within half an hour of scanning and are already used in many conventional vision studies. We used these responses to estimate the pRF-properties of each cortical location [3]. Next, we measured fMRI responses elicited by both synthetic stimuli and natural images. Synthetic images are widely used because they are more easily controlled. However, responses to synthetic images may not extrapolate to natural images [9–12]. Therefore, by using both synthetic and natural images, we aim to bridge the gap between experimental settings and real-life situations. We identified the presented image based on the similarity of the measured fMRI signals to the predictions of the pRF-model. Using the voxels of visual field map V1, image identification from large image datasets (1000 synthetic stimuli or 200 natural images) is far above chance, both for the synthetic and natural images. As such, we show that it is possible to identify natural images using a pRF-model with minimal parameters.

## Methods

### Subjects

Two subjects (one female; ages 28–38 years) participated in this study. All subjects had normal or corrected-to-normal visual acuity. All studies were performed with the informed written consent of the subjects and were approved by the Human Ethics Committee of University Medical Center Utrecht.

### Stimulus presentation

The visual stimuli were generated in Matlab using the PsychToolbox [13, 14] on a Macintosh Macbook Pro. The stimuli were back-projected on a display inside the MRI bore. The subject viewed the display through mirrors inside the scanner. The size of the display was 15.0x7.9 cm with a resolution of 1024x538 pixels. The total distance from the subject’s eyes to the display was 41 cm. The stimuli were constrained to a circular area with the size of the vertical dimension of the screen. The area outside this circle was remained at a constant mean luminance. This gave the stimulus a radius of 5.5° visual angle from the subject’s point of view.

### Stimuli used to estimate pRF properties

**Fig 1:**
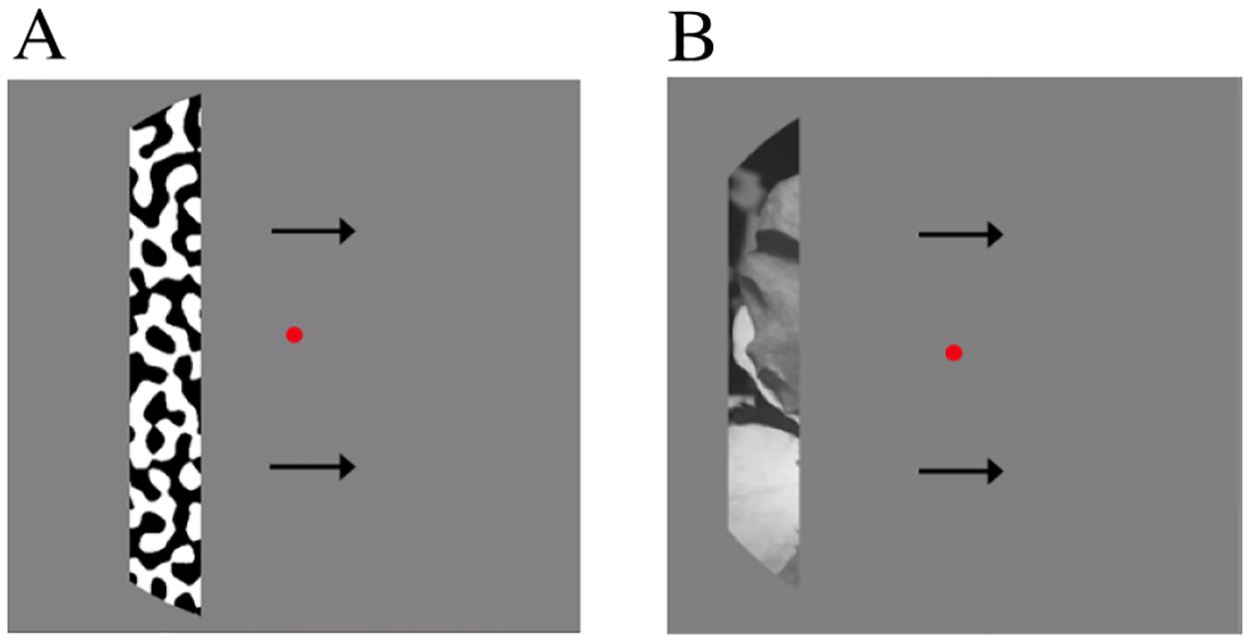
Examples of the pRF mapping stimuli used to estimate the pRF properties. The stimuli were either filled with a binarized bandpass filtered noise pattern (A) or natural image content (B).

The pRF properties were estimated using conventional contrast-defined moving-bar apertures (Fig 1) [3]. The width of the bar subtended 1/4^th^ of the stimulus radius (1.375°). Four bar orientations (0°, 45°, 90° and 135°) and two different step directions for each bar were used, giving a total of 8 different bar configurations within a given scan. The bar stepped across the stimulus aperture in 20 steps (each 0.55° and 1.5 seconds) with each pass taking 30 seconds. A period of 30 seconds mean-luminance (0% contrast) was presented after every horizontally or vertically oriented pass. In total there were 4 blocks of mean-luminance during each scan, presented at evenly spaced intervals.

We used two different sets carrier-images within the moving bar apertures. The bar apertures used to train the pRF-model to identify the synthetic images were filled with a binarized bandpass filtered noise pattern (fundamental frequency is 1.5 cycles/deg). This pattern was presented with three different alternating high-contrast patterns, to obtain a full high-contrast response that is not based upon one specific high-contrast pattern. The bar apertures used to train the pRF-model to identify the natural images contain natural image content (Fig 1). Each bar aperture was presented for one TR (1.5s). For each bar aperture three different natural image carriers were used. The natural image content was replaced every 500ms. The image was shown for 300ms with a 200ms mean-luminance gap.

The natural image content for the bars came from images of the 'Berkeley Segmentation Dataset and Benchmark' database [15]. The synthetic and natural images used in these bars did not include those used in the image identification test sets.

The subjects performed a fixation dot task to make sure they fixated at the center of the display. A small fixation dot (0.11° radius) was presented in the middle of the stimulus. The fixation dot changed its color from red to green at random time intervals and subjects were instructed to respond at this change of color using an air pressure button (average performance of 93.3%).

### Stimuli used for image identification

For the image identification process we used two different sets of images. We used both synthetic images and natural images. The synthetic images were scanned for one subject only, as a proof of concept for the image identification process. The images were presented in a block design. Each image was presented during a 9-second block. Within this block the same image was shown 18 times for a duration of 300ms followed by 200ms mean-luminance. The block where the image was presented was followed by a 12 second mean-luminance presentation. The synthetic images were presented with 3 alternating different high-contrast patterns, to obtain a full high-contrast response that is not based upon one specific high-contrast pattern. A fixation dot was presented at the center of the stimulus in both stimulus sets. The same fixation dot task as in the scans of the mapping stimuli was used (average performance of 91.5%).

#### Synthetic images

**Fig 2:**
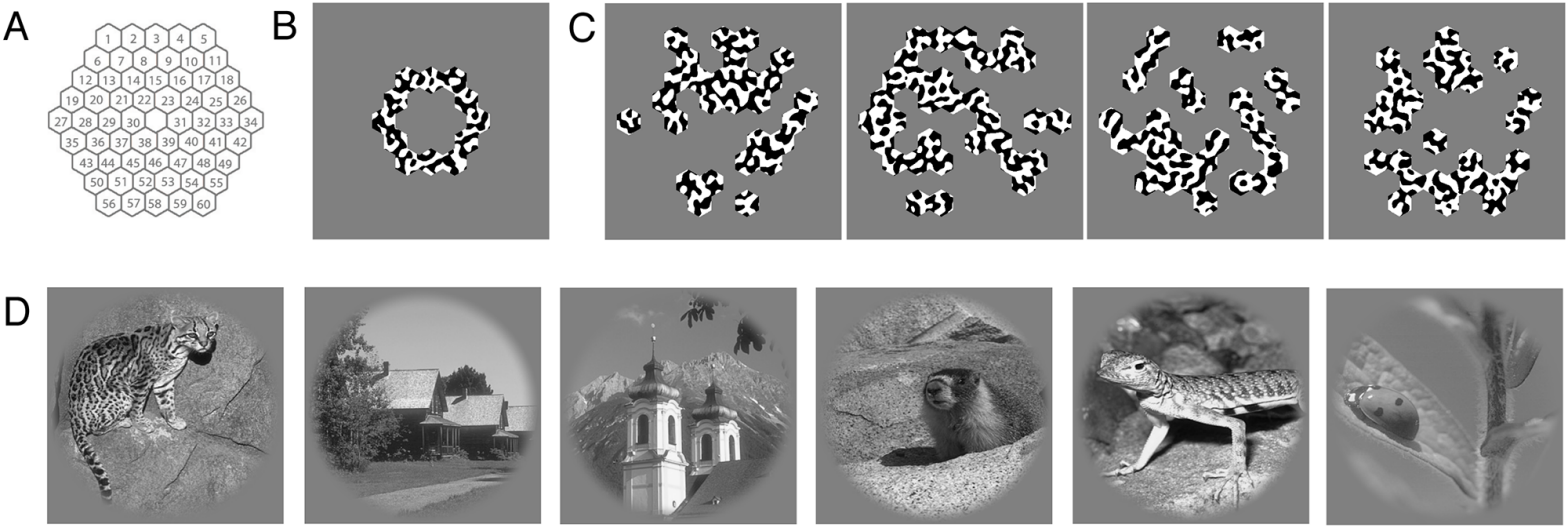
Image identification stimuli. (A) The synthetic stimuli were made using a grid of 60 hexagons (cells) that could be filled with either binarized bandpass-filtered noise or mean-luminance gray. Examples of the stimuli used in the experiment; (B) a non-random synthetic stimulus, (C) random synthetic stimuli and (D) natural images.

The synthetic images were semi-randomly generated patterns consisting of a hexagonal grid partially filled with binarized bandpass-filtered noise. The grid contains 60 small hexagons (cells), of which a random selection were filled with binarized bandpass filtered noise, and the rest filled with mean-luminance gray. This random selection gives 2^60 (>> trillion) possible images. We used 3 sets of these images in different scanning runs, with each set containing 15 different synthetic images (45 in total) and one full-field binarized bandpass-filtered noise stimulus. Fig 2B–2C shows an example of the synthetic images used in the experiment. We used 5 non-random patterns (Fig 2B) and 40 random patterns (Fig 2C), they were combined in the image identification process.

#### Natural images

The natural images for the image identification process came from the 'Berkeley Segmentation Dataset and Benchmark' database [15]. The original resolution of the images was 321x481 pixels (both landscape and portrait). We selected a squared part of 321x321 pixels from the images and upsampled this to a resolution of 538x538 pixels, which corresponds to a stimulus of 11x11° diameter of visual angle. The images were masked by a circle with a raised cosine faded edge (width of 0.9°), and the areas outside this circle were set to mean luminance. The images were gamma-linearized and the mean contrast was set to 50%. We used 3 image sets in different scanning runs, each containing 15 different natural images (45 in total) and one full-field binarized bandpass-filtered noise stimulus. Fig 2D shows an example of the natural images.

### Functional imaging and processing

The MRI data was acquired with a Philips 7T scanner using a 32-channel head-coil. We scanned the participants with a 2d-echo-planar-imaging sequence with 26 slices oriented perpendicular to the Calcarine sulcus with no gap. The following parameters were used; *repetition time* (TR) = 1500 ms, *echo time* (TE) = 25 ms and a flip angle of 80°. The functional resolution was 2x2x2 mm and the *field of view* (FOV) was 190x190x52 mm.

We used foam padding to minimize head movement. The functional images were corrected for head movement between and within the scans [16]. For computation of the head movement between scans, the first functional volumes for each scan were aligned. Within scan motion correction was then computed by aligning the frames of a scan to the first frame.

The duration of the pRF mapping scans was 372 seconds (248 time-frames), of which the first 12 seconds (8 time-frames) were discarded due to start-up magnetization transients. During the three sessions for the synthetic images we acquired 10 pRF mapping scans in total. For the natural image sessions we scanned 2 subjects and acquired during the three scanning sessions 6 or 7 pRF mapping scans in total per subject. To obtain a high signal-to-noise ratio, we averaged the repeated scans. The duration of the image identification scan (both synthetic and natural) was 432 seconds (288 time-frames). The first 12 seconds (8 time-frames) were discarded due to start-up magnetization transients.

During the three sessions for the synthetic images we acquired three scans for each of the three different image sets. During the three sessions for the natural images we acquired two scans each for the three natural image sets.

### Anatomical imaging and processing

The T1-weighted MRI images were acquired in a separate session using an 8-channel SENSE head-coil. The following parameters were used: TR/TE/flip angle = 9.88/4.59/8. The scans were acquired at a resolution of 0.79x0.80x0.80 mm and were resampled to a resolution of 1mm^3^ isotropic. The functional MRI scans were aligned with the anatomical MRI using an automatic alignment technique [16]. From the anatomical MRI, white matter was automatically segmented using the *FMRIB's Software Library* (FSL) [17]. After the automatic segmentation it was hand-edited to minimize segmentation errors [18]. The gray matter was grown from the white matter to form a 4 mm layer surrounding the white matter. A smoothed 3D cortical surface can be rendered by reconstruction of the cortical surface at the border of the white and gray matter [19].

## pRF model-based analysis

**Fig 3:**
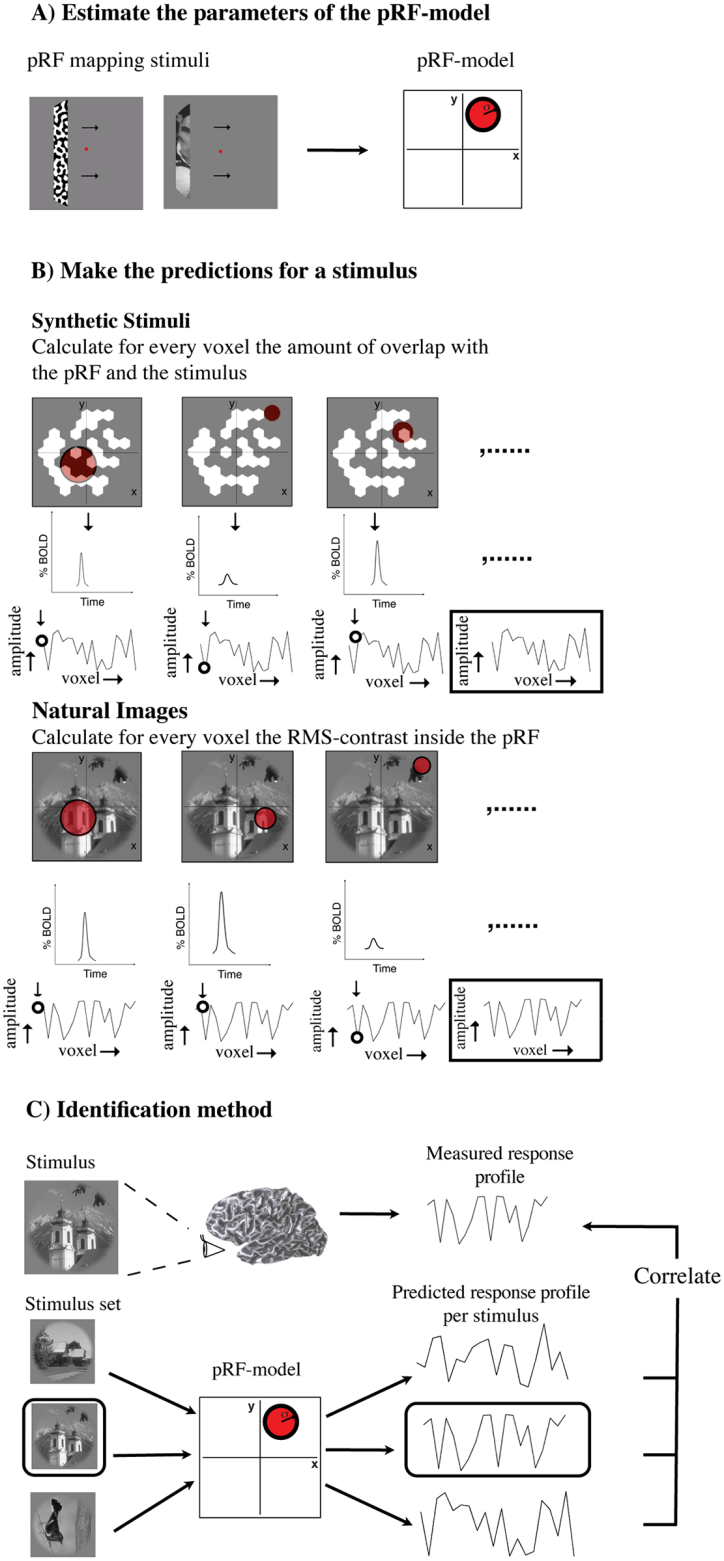
Schematic diagram of the image identification pipeline. A: First, we estimated the parameters of the pRF for every voxel based on the pRF bar stimuli. The pRF is modeled as a circular symmetric Gaussian function of which we use the parameters for position and size. B: Second, we predicted the response profiles for a large set of candidate images by either summing the overlap of the stimulus with each voxel’s pRF (for the synthetic images), or by calculating the RMS-contrast inside each voxel’s pRF (for the natural images). C: Finally, we predicted which candidate image elicited a measured response profile by finding which candidate image’s predicted response profile was most strongly correlated to this measured response profile (i.e. which had the highest pearson’s *r*).

The first step for image identification is the estimation of the pRF-model from the measured fMRI signal that was elicited by the pRF mapping bar stimuli (Fig 3A). The model estimates a pRF for every cortical location using a method previously described [3]. For technical and implementation details see [3] but in short, the method estimates the pRF by combining the measured fMRI time-series with the position time course of the visual stimulus. A prediction of the time-series is made by calculating the overlap of the pRF and the stimulus energy for each time frame convolved with the haemodynamic response function (HRF). We estimated the parameters of the HRF that best describes the data of the whole acquired fMRI volume [20]. The optimal parameters of the pRF-model are chosen by minimizing the residual sum of squares between the predicted and the measured time-series. We used the simplest pRF-model which consists of a circular symmetric Gaussian. This model has four parameters: position (x_0_, y_0_), size (σ_1_), and amplitude (β_1_).

Using the pRF-method, we estimated position parameters x_0_, and y_0_ of the pRF per voxel. From these values, the polar angle (atan(y_0_/ x_0_)) and eccentricity (√(x_0_^2^ + y_0_^2^)) values can be calculated. We drew the borders of the visual areas on the basis of their location in the visual field [21–24] by rendering these polar angle and eccentricity maps onto the inflated cortical surface [19]. We defined visual area V1, V2 and V3 as our *region of interest* (ROI).

### Image identification

#### Analysis of signal responses elicited by viewing synthetic and natural images

We measured fMRI responses to 45 synthetic images and to 45 natural images. We first determined the distribution of the responses at each recording site (voxel) elicited by each of these images. To estimate the responses we used standard GLM-analysis [25, 26]. Briefly, we fitted a block-design to the stimulus presentation convolved with the HRF. We summarized every voxel’s response by its t-value, which reflects the goodness of fit between the predicted time series and the measured data for each cortical location for that image. For image identification, we only used voxels with positive t-values, pRF eccentricity values from 0.5–4.5° and pRF variance explained above 55%.

#### Prediction response profiles for synthetic and natural images using the pRF model

In the identification process, we had a large candidate set of images from which to choose the image that was presented. To do this, we compared the measured response profiles against the response profiles predicted by the pRF-model. We determined these predicted response profiles from the pRF-model as follows (Fig 3B).

We converted the synthetic images to binary images where cells filled with bandpass-filtered noise were set to a binary value of 1 and cells filled with mean-luminance gray were set to 0. The prediction response profile is calculated by the summed overlap of the stimulus with the pRF of each cortical location and is normalized by the total volume of the pRF:

$$voxelprediction\_to\_synthetic\_image=\frac{\sum_{i=1}^{N}w_{i}\cdot S_{i}}{\sum_{i=1}^{M}w_{i}}$$ (1)

Where N is the number of pixels in the spatial window of the pRF and M is the total number of pixels in the stimulus area. S is the binary stimulus, where S_i_ is the binary value of whether the pixel of the stimulus was on (1) or off (0). The pRF weighting function is defined by *w*_*i*_:

$$w_{i}=\text{exp}-\left(\frac{{\left(x_{i}-x_{c}\right)}^{2}+{\left(y_{i}-y_{c}\right)}^{2}}{2{\left(\sigma \right)}^{2}}\right)$$ (2)

Where x_c_ and y_c_ define the location of the center of the pRF in the visual field, σ is the size of the pRF and x_i_ and y_i_ define the location of the *i*th pixel.

For the natural image we followed a slightly different approach as the stimulus cannot be binarized in the same way as the synthetic images. We predicted each voxel’s response to each candidate natural image by calculating the Root-Mean-Squared (RMS) contrast [14, 27] weighted by the corresponding pRF. RMS contrast is defined as the standard deviation of the luminance intensities relative to the mean. The RMS-contrast is weighted by the pRF-function to obtain the local contrast-energy value per pixel:

$$local\_contrast\_energy=\sqrt{\frac{1}{\sum_{i=1}^{N}w_{i}}}\overset{N}{\underset{i=1}{\sum }}w_{i}\frac{{\left(L_{i}-L\right)}^{2}}{L^{2}}$$ (3)

Where N is the number of pixels in the spatial window of the pRF. L is the mean luminance from the pixels inside the spatial window, and L_i_ is the luminance of the *i*th pixel.

The predicted response profile for each candidate image consisted of the predicted response amplitudes of all voxels within a given visual area. These response amplitudes are not further convolved with the HRF, as this would yield a linear amplitude transformation that leaves relative response amplitudes within the overall response profile intact.

#### Correlating predicted with measured responses profiles

To identify the image that was shown to the subject we compared the measured response profiles elicited by the presented image (Im_i_) to the predicted response profiles for each candidate image {Im_1_, Im_2_, Im_3_, …, Im_n_}. The presented image was identified by choosing the candidate image that gives the highest correlation (Pearson’s *r*) between its predicted and the measured response profile (Fig 3C).

We calculated the percentage of correct image identifications within each set of 15 images. We also increased the set size of candidate images by including images that we never showed. We increased the set of candidate images to 1000 for synthetic images, and to 200 for natural images. This makes correct image identification by chance less likely. We bootstrapped this analysis for each set size, by making 1000 different combinations of different candidate images. From resulting proportions of correct image identifications among these candidate image sets, we computed the mean image identification performance and the 95% confidence interval of this mean.

## Results

### Successful image identification for the synthetic images

**Fig 4:**
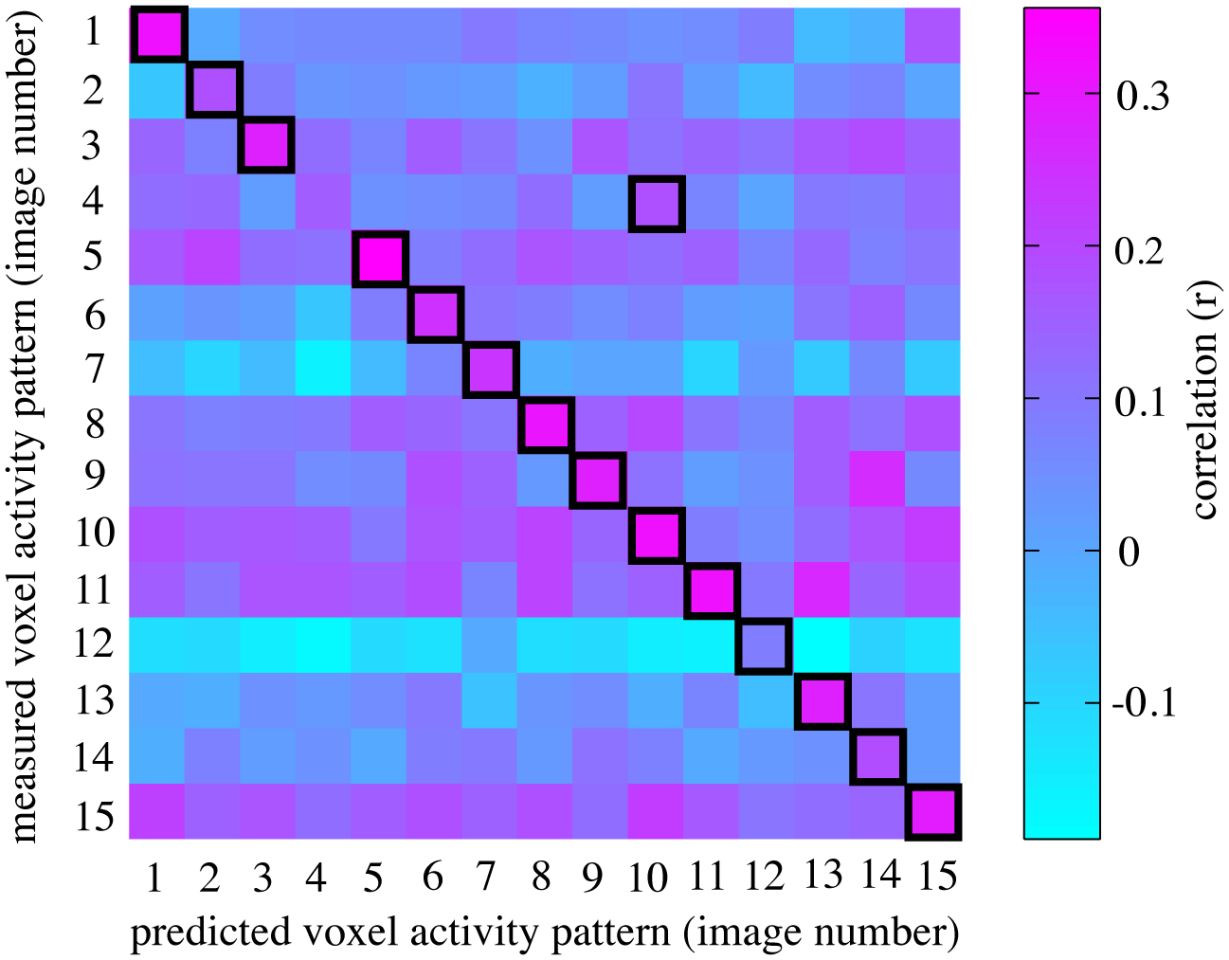
The correlation matrix for an example set of synthetic images. The correlation matrix shows the prediction accuracy using the voxels of V1 from the image identification process for an example set of the synthetic images. The colors represent the correlation (Pearson’s *r*) of the measured response profiles from all the images with their predicted response profile (from the pRF-model). For this image set, 14 out of 15 images were identified correctly, giving a prediction accuracy of 93.3%.

To demonstrate that images could be identified using pRF-models, we first performed a proof of concept experiment using synthetic, high contrast images in a single subject. We identified the synthetic images using the pRF-model that summarized responses to bar apertures that revealed binarized bandpass filtered noise (Fig 1A). We used this model to predict the response profiles for a set of possible candidate images. We then generated a correlation matrix by correlating the predicted response profiles for every image to the measured response profiles. Fig 4 shows the correlation matrix for the voxels of primary visual cortex (V1) for one of the stimulus sets. The y-axis gives the correlation values of the stimulus set’s measured response profiles to the predicted response profiles (on the x-axis). The black outlines show the candidate image whose predicted response profile was most highly correlated to the measured response profile, which we predicted was the presented image. The prediction accuracy for this subject was 93.3% (14 out of 15).

**Fig 5:**
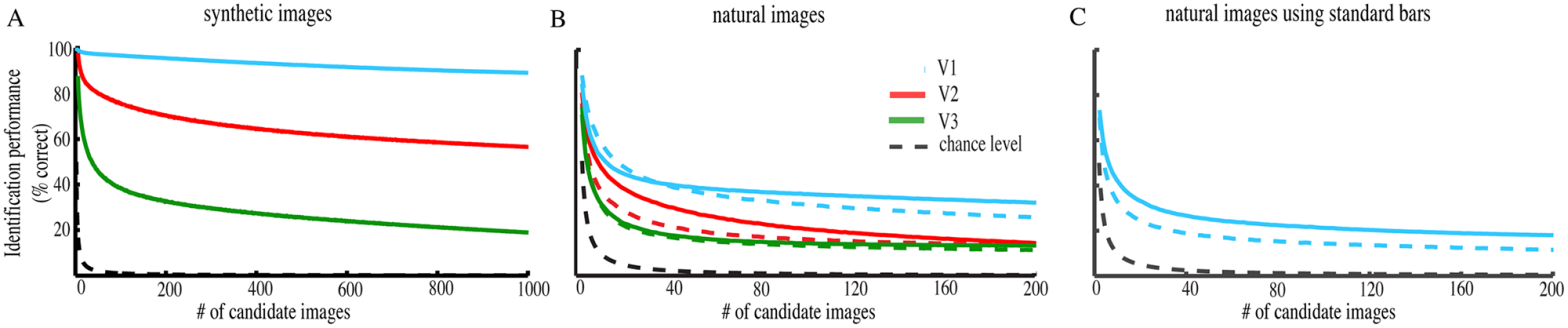
Image identification performance both for synthetic (A) and natural image stimuli (B, C). The pRFs were estimated in a separate scan where the bars were filled with synthetic (A, C) and natural (B) stimuli either in the same session (A,B) or in different sessions on different days (C). The blue, red and green lines show the performance for visual field maps V1, V2 and V3 respectively, for 2 subjects (closed and dashed colored lines). The thickness of the lines includes the 95% confidence intervals. For the synthetic image stimuli we increased the set size up to 1000 different images, for the natural images we increased the set size up to 200 different images. The black dashed line indicates the chance level. The high-contrast synthetic images were most accurately identified (A), and the natural images were also identified far above chance for all candidate image set sizes (B). Furthermore, the identification of the natural images is also possible with the pRF-model that was estimated using standard bar stimuli containing moving checkerboards, and estimated from a separate scanning session on a separate day (C).

The correlation matrix shows the prediction accuracy using only the predicted response profiles for images within the presented set, giving the image identification performance a chance level of 1/15 images (chance level = 6.7%). Using the pRF-model, we can make predicted response profiles for any image, including those that were never presented. This makes correct image identification less likely, and more accurately quantifies the likelihood of identifying the correct image by chance. Fig 5A shows the performance when we make predicted response profiles for up to 1000 different randomly generated synthetic stimuli. This reduces the chance level to 1/1000 (chance level = 0.1%). Nevertheless, the correct image was still identified correctly from 1000 candidate images for 89.0% of the presented images for visual area V1. The performance drops for later visual areas V2 (~56%) and V3 (~18%), but still remains high above chance. The thickness of the line includes the 95% confidence intervals, determined by bootstrapping the mean.

### Successful image identification for natural images

Next, we extended this approach to natural images. To predict response profiles for the natural images, we used the pRF-model that summarized responses to bar apertures containing natural image content (Fig 1B). The natural image content in these bars was independent from the natural images used for the image identification process. We scanned 45 different natural images for two subjects. For the 45 images the performance is 33% (15 out of 45 images) both for subject 1 and 2, the performance for a random selection of 45 images is ±38%. Both are very different from chance (2.2%). We choose to show the correlation with a larger set-size because idiosyncrasies of the chosen set size of 45 images are removed and results are more generalizable.

Fig 5B shows the identification performance for the natural images for both subjects (closed and dashed colored lines) as a function of candidate image set size, up to 200 candidate natural images. The thickness of the lines includes the 95% confidence intervals, determined by bootstrapping the mean. In each subject, image identification performance was far above chance, with approximately 29% correct image identification performance with 200 candidate images (chance level = 0.5%). The identification performance for visual areas V2 (~14%) and V3 (~12%) is lower compared to V1, but remains far above chance.

Besides the pRF-model that was estimated using bars with natural image content, we also used a pRF-model that was estimated on the standard pRF mapping stimulus consisting of contrast-defined bar apertures containing moving checkerboards (Fig 5C) [3]. The latter stimulus is widely used to model pRF properties [28–44]. In addition, the pRF estimates were acquired on different days as the natural image data-set, as many researchers already have such pRF models. In these circumstances, we still find that it is possible to identify natural images above chance level (~15%).

### Image identification accuracy depends on image content

**Fig 6:**
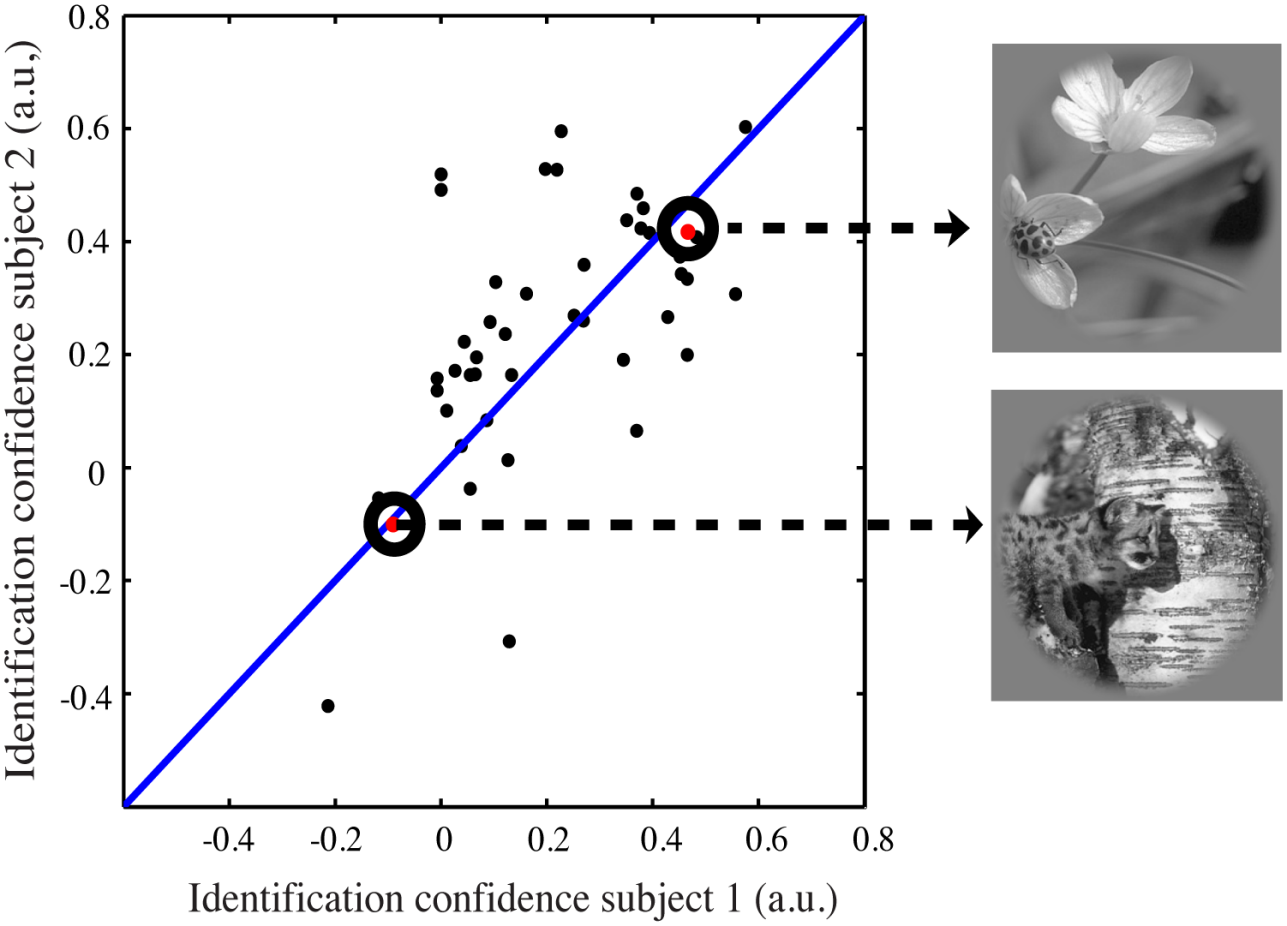
The identification confidence per natural image for our two subjects. Every dot represents an individual image. We see similar confidence of the individual natural images across the two subjects. Some images are identified less accurately using the pRF-model than others. This is explained by two factors: (i) certain images are more similar in terms of their contrast-energy content and (ii) responses to these images depend more on features that are not captured by the contrast-energy pRF-model predictions.

We examined image identification accuracy for individual natural images. This reveals which images are most and least accurately identified. Fig 6 shows identification confidence for each image, based upon the mean difference of the correlation score of the presented image and all other candidate images.

$$confidence\_score_{i}=\frac{-\sum_{j=1}^{N}corr(i_{m},j_{p})-corr(i_{m},i_{p})}{N}$$ (4)

Where *i* is the image to calculate the score for, corr(*i*_*m*_,*j*_*p*_) is the correlation score (pearson’s *r*) of the measured (*m*) response profile of the *i*th image and the predicted (*p*) response profile of the *j*th image. corr(*i*_*m*_,*i*_*p*_) is the correlation score of the measured and predicted response profiles of the presented image and N are the number of images in the set. The confidence score represents how hard it is to distinguish the presented image from other candidate images. A high difference in correlation scores will give a high confidence score. Here we see that for both subjects the same images are harder to identify than other images. We find a significant correlation (Pearson’s *r*) of *r*(43) = 0.58, p<<0.001.

Images could be harder to identify because they are more similar to other images or the visual representation deviates more from their contrast-energy content. To distinguish these possibilities, we checked the images for image similarity in contrast-energy using the predicted response profiles of the pRF-model. We found a significant effect for similarity in contrast between the images and how well we were able to predict the presented image. Thus images that were more similar in contrast-energy information as the other images in the candidate set of images to choose from were harder to identify. Nevertheless, after removing the relation with contrast (using GLM-analysis), we still found the effect across subjects that the same images were harder to identify than other images (r = 0.53 p << 0.001). This suggests that image performance for certain images is deteriorated because both (i) certain images are more in terms of contrast information and (ii) the measured responses of certain images hold information about image features that are not captured by the pRF-model.

## Discussion

We describe an effective method to identify presented natural images from the fMRI responses of V1 voxels using pRF-models. We used this method to identify both synthetic and natural images with accuracy far above chance. The synthetic images were easier to identify than the natural images, suggesting that image identification performance is affected by the spatial distribution of contrast within the identified images. The pRF model we employed had minimal parameters (3) and was estimated with very different stimuli. Even when using a pRF-model derived from a different scanning session and using the standard moving checkerboard stimuli, it is possible to identify natural images. This allows the image identification method to be more broadly applied.

We believe that this method is generally applicable despite our small sample size. First, previous papers have demonstrated image identification using similar approaches tested over small sample sizes. For example Kay et al. also use 2 subjects [4, 5]. Second, we have shown successful image identification in each subject with very high statistical confidence. Data from further subjects may show better or worse on image identification performance, but we believe this will reflect differences in data acquisition quality rather than reflecting any methodological limitation. Third, while conventional fMRI studies average data across large subject numbers, most collect less data from each subject. Our approach is to collect a large dataset for a small number of subjects and to analyze our data per subject in a voxel-wise modeling approach. The advantage of this approach is that detailed spatial information and individual variability is retained. This information is lost in the conventional fMRI studies by averaging of the data across subjects.

Image identification was possible for both the synthetic and natural images. In both cases, identification accuracy was far above the chance level. However, the identification accuracy was higher for synthetic than natural images. There are several possible reasons for these differences. The synthetic images were simple binary patterns of high contrast, designed to elicit a strong fMRI responses. Natural images have a broader spectrum of lower contrasts, and so elicit lower amplitude fMRI responses, with a lower signal to noise ratio (t-test on %BOLD signal change of the natural vs. synthetic images: t(85379) = 26.73, p<<0.01).

Furthermore, pRF-models as employed here only represents contrast energy. Early visual cortex is known to respond very strongly to differences in contrast [45–49]. The synthetic images are dominated by their contrast content, so responses to synthetic images are better captured by the pRF model than responses to natural images. While the synthetic images contained all orientations and spatial frequencies within any local area, natural images had highly variable spatial distributions of both orientation and spatial frequency. Orientation content is known to affect the responses of each voxel [4, 50–52], and spatial frequency content is also likely to affect voxel responses [4, 48, 53, 54]. Our pRF models do not account for these factors. Furthermore, responses to natural images are also likely to be affected by high-level image features and global image context [55], which are also not captured by the pRF-model.

Also, where image identification was possible using the voxels of visual areas V1, V2 and V3, we see that the accuracy drops for the later visual areas V2 and V3. The reason for this drop in accuracy can have several reasons. First, pRF sizes get bigger in higher visual areas [3, 56]. This increase in receptive field sizes might decrease the resolution necessary for image identification. Second, the visual field maps get smaller for later visual areas [57], giving less measurement sites to perform the analysis. Third, the pRF-model only captures the contrast energy. As described above, other features of the images can be captured in the measured responses that are not represented by the responses predicted using the pRF-model. This effect can be bigger for later visual areas that are proposed to respond to more complex features [58, 59].

Fig 6 shows the identification confidence of the individual natural images for both subjects for visual area V1. We see that some images are identified with less confidence than others and that these images are similar across subjects. We propose that this is due to two reasons. First, images that are more similar in terms of their content in contrast-energy are harder to distinguish. Second, responses to images that are harder to identify depend more on image content that is not captured by the pRF model, as discussed above. Differences in identification confidence can also indicate differences in the perception of images across subjects. This method can potentially be used to investigate these differences.

The pRF model we employ represents the most basic encoding model. Encoding models give information about how the information is represented in the voxels [3–8, 60, 61]. These models use biologically inspired computations that make a translation between the stimulus and the response. The pRF model uses two parameters for visual field position (x and y) and a third for pRF size. PRF position and size are inherently necessary here, since we calculate the contrast within a specified area of the image to predict the response of each recording site. This area must have a size because a single image location (pixel) has no contrast. Since encoding models predict the activation pattern in response to a certain stimulus, these models can be inverted to decode and hence can be used for the purpose of image identification.

Alternatively, decoding methods define a categorical relationship between the activation patterns and the stimulus, and can be used to indicate whether a voxel contains information about a certain property of a (visually) presented stimulus [1, 2, 50, 51, 62, 63]. However, since they do not give a further description of how this information is represented in the voxels, these models are only able to make a prediction about the predefined categories that the model was explicitly trained on. It is not possible to make a prediction about any other property of the stimulus. This makes these models unsuitable for image identification, where every possible image should be able to be identified.

Last, both decoding and encoding models can reconstruct the image from the fMRI signal. The goal of image reconstruction is to reproduce the presented image. Reconstructions have been made for simple high contrast patterns [5, 64], imagined [5] and illusory contours [60], dreams [65] as well as natural images [6]. The studies of Thirion et al., 2006 and Miyawaki et al., 2008 also used the reconstructions to perform synthetic image identification by comparing the reconstructed image with a set of candidate images. The study of Naselaris et al., 2009 used their image reconstructions to select images that were most similar to the presented image in terms of image structure and semantic content.

In the study of Kay et al., 2008, they use an encoding model of the visual system to identify natural images. They model the internal representation of the visual cortex using a Gabor-Wavelet-Pyramid (GWP). The GWP has parameters for position, orientation and spatial frequency. The Gabor filters of the model are applied to an image, and the combined outputs of the filters make the response prediction for that image. The main advantage of this approach (and for the pRF-model) is that it can predict the activation pattern for any image.

Using the GWP for image identification instead of the pRF-model leads to a better identification performance [4]. Several differences between these studies may account for performance differences. First, Kay et al presented images for 1 second at a time, while we presented the images for 9 seconds. Longer presentations might lead responses to reflect more influences from higher-level image representations, which neither model represent. Second, Kay et al. used larger images (20x20° of visual angle) than we did (11x11°). Larger images would cause responses in a larger extend of visual cortex, which provides more data for identification. Third, the models differed greatly in their complexity. Where the standard pRF model uses only 3 free parameters to capture each recording site’s response, the GWP uses up to 2,370. Kay et al. also included a retinotopic-only (RO)-model for image identification. In this model they removed the parameters for orientation and spatial frequency. Therefore, this model has fewer parameters compared to the original GWP. This model is conceptually similar to the pRF-model we use, but there are important differences. First, our pRF-model describes the pRF using one single circular Gaussian, while the RO-model is free in the shape of the responsive area. Second, the RO-model therefore has more free parameters than our pRF model. More parameters in the model will allow more accurate representation of voxels responses, but the drawback of using a model with a large amount of free parameters is that a large dataset has to be used to train the model. The estimation process of the model uses the fMRI data elicited by the example images of the training set. This means that for using models with more free parameters, more scanning time is needed as well. The GWP was estimated using a set of 1,750 different natural images. To obtain the data for estimating the GWP takes about 5 hours. To compare this with the pRF-model, this was estimated using standard mapping stimuli with 81 differential images, which only take about half an hour of scanning.

## Conclusion

The pRF-method is a fast and simple biologically inspired model. We show that, even when training it on conventional pRF mapping stimuli, it can be used for the identification of visual images, including natural images. The advantage of using the pRF-method for image identification is that the model has a minimal amount of free parameters. Collecting the fMRI data for the estimation of the pRF-model can be done within half an hour of scanning using standard mapping stimuli. This makes the pRF-model a convenient method to be used for the identification of untrained images, including natural images.
